# Freshwater snail-borne parasitic diseases in Africa

**DOI:** 10.1186/s41182-024-00632-1

**Published:** 2024-09-20

**Authors:** Papa Mouhamadou Gaye, Souleymane Doucouré, Doudou Sow, Cheikh Sokhna, Stéphane Ranque

**Affiliations:** 1 Aix-Marseille Université, IRD, AP-HM, SSA, VITROME, 13005 Marseille, France; 2 VITROME, Campus International IRD-UCAD de L’IRD, 1386 Dakar, Senegal; 3 Institut Hospitalo-Universitaire (IHU) Méditerranée Infection, 19-21 Bd Jean Moulin, 13005 Marseille, France; 4 Département Biologie Animale, Faculté Des Sciences Et Technique, UCAD, 5005 Dakar, Senegal; 5 Department of Parasitology-Mycology, UFR Sciences de La Santé, Université Gaston Berger, 234, Saint Louis, Senegal

**Keywords:** Schistosomiasis, Fasciolasis, Freshwater, Snail, Parasite, Diseases, Africa

## Abstract

**Background:**

Freshwater snails are the first obligatory intermediate hosts in the trematode life cycle. Several parasitic diseases transmitted by these snails are endemic in Africa, and their distribution closely follows that of the intermediate hosts. These diseases represent a major public health problem and cause significant socio-economic losses in Africa, particularly schistosomiasis and fascioliasis. In this review, we will describe the main roles of freshwater snails in the life cycle of trematode parasites, and the geographical distribution of these diseases in Africa. We will also discuss the different techniques for detecting parasitic infections in snails, as well as the various methods of controlling snails and the larval stages of parasites.

**Methods:**

We carried out a literature search for articles dealing with parasitic diseases transmitted by freshwater snail hosts in Africa. The search was conducted in databases such as PubMed, Web of Science and Google Scholar using various search terms combined by Boolean operators. Our search was limited to peer-reviewed articles less than 10 years old. Articles published to date in the fields of control of parasitic diseases transmitted by freshwater snails were included. Results were presented in narrative and in table format.

**Results:**

The results of the database search identified 1007 records. We included 84 studies in this review. These studies generally focused on freshwater snails and the diseases they transmit. We described the geographical distribution of 43 freshwater species belonging to nine snail families, as well as the parasites that infect them. Several methods for diagnosing parasites in their snail hosts have been described, including microscopic and molecular methods, as well as antibody and protein barcode-based techniques. Molluscicides have been described as the main strategy for snail control.

**Conclusion:**

This study highlights several elements of knowledge about diseases transmitted by freshwater snails and their distribution. A good understanding of snail infection detection techniques and existing control methods is an essential component in adapting control strategies for these diseases.

**Graphical Abstract:**

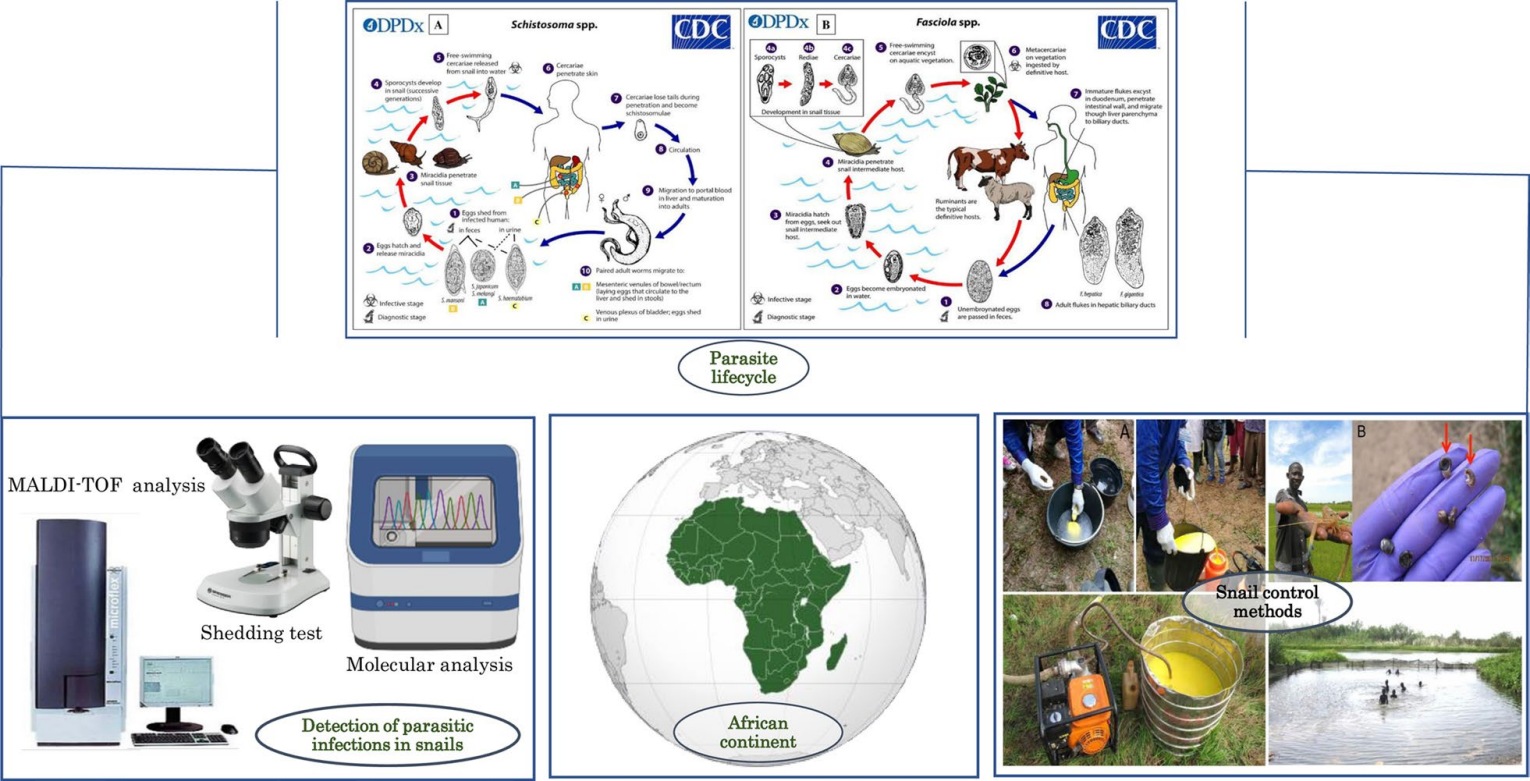

**Supplementary Information:**

The online version contains supplementary material available at 10.1186/s41182-024-00632-1.

## Introduction

Freshwater snail-borne parasitic diseases (FSBPDs) represent a major public health problem worldwide, particularly those caused by trematodes such as schistosomiasis and fascioliasis [1]. Both these trematodiasis are considered as neglected tropical diseases (NTDs) by the World Health Organization (WHO) [1]. These FSBPDs pose a risk to human health, affecting millions of people and causing major socio-economic losses, particularly in poor African populations. Freshwater snails serve as obligatory intermediate hosts in the lifecycle of parasites and play a major role in the epidemiology of trematodiasis, notably schistosomiasis and fascioliasis.

Schistosomiasis is the second most important endemic parasitic disease after malaria, in terms of its impact on public health. It affects more than 250 million people worldwide, including children and young people, and is responsible for almost 200,000 deaths a year [1].

The disease is most prevalent in low-income countries, particularly in sub-Saharan Africa (SSA) [1, 2]. In these regions, prevalence is particularly linked to irrigation systems, agricultural activities [3] and poor socio-environmental conditions, including lack of drinking water. All these factors allow permanent contact between humans and snails, contributing to maintain transmission [4]. The disease involves various trematodes of the genus *Schistosoma* [5], with snails of the genera *Biomphalaria* and *Bulinus* serving as intermediate hosts for their larval development. The most widely known are *Schistosoma haematobium*, *S. mansoni*, *S. intercalatum* and *S. guineensis* [6, 7].

Fascioliasis is a liver disease of domestic livestock caused by infestation with flukes of the *Fasciola* genus [8]. It is a worldwide zoonotic infection common to ruminants and present in more than 70 countries, particularly where sheep or cattle are reared [9]. It is widely distributed in tropical and sub-tropical areas of Africa and Asia, where it has a major impact on the productivity of domestic ruminants [10, 11]. Fascioliasis is a near-cosmopolitan zoonosis, with sporadic cases in humans occurring in most parts of the world. Human fascioliasis is currently classified among food/plant trematode zoonoses as an NTD [9]. Human fascioliasis also causes significant illness and morbidity, mainly in low-income farming communities. It is estimated that more than two million people worldwide are infected [12]. *Fasciola gigantica* and *F. hepatica* are the main trematodes and can infect a wide variety of domestic animals, wild animals and humans [11]. Several snail species found in Africa, notably *Lymnaea natalensis* and *L. trunctula*, play an essential role in the transmission of trematode infections such as fascioliasis [13, 14].

This is followed by the transmission of parasitic diseases, which is highly dependent on the expansion of intermediate hosts and the rural development of water resources. The study of freshwater snail vectors provides vital information on the active transmission foci of parasitic infections. However, few studies have focused on the crucial role of freshwater snails in the transmission of parasites. Hence our review of FSBPDs in Africa, will focus on two major diseases (schistosomiasis and fascioliasis). We will focus on the geographical distribution of these parasitic diseases and their intermediate hosts, the detection of parasites, and the control of snail vectors.

## Materials and methods

### Search strategies and inclusion criteria

**Fig. 1:**
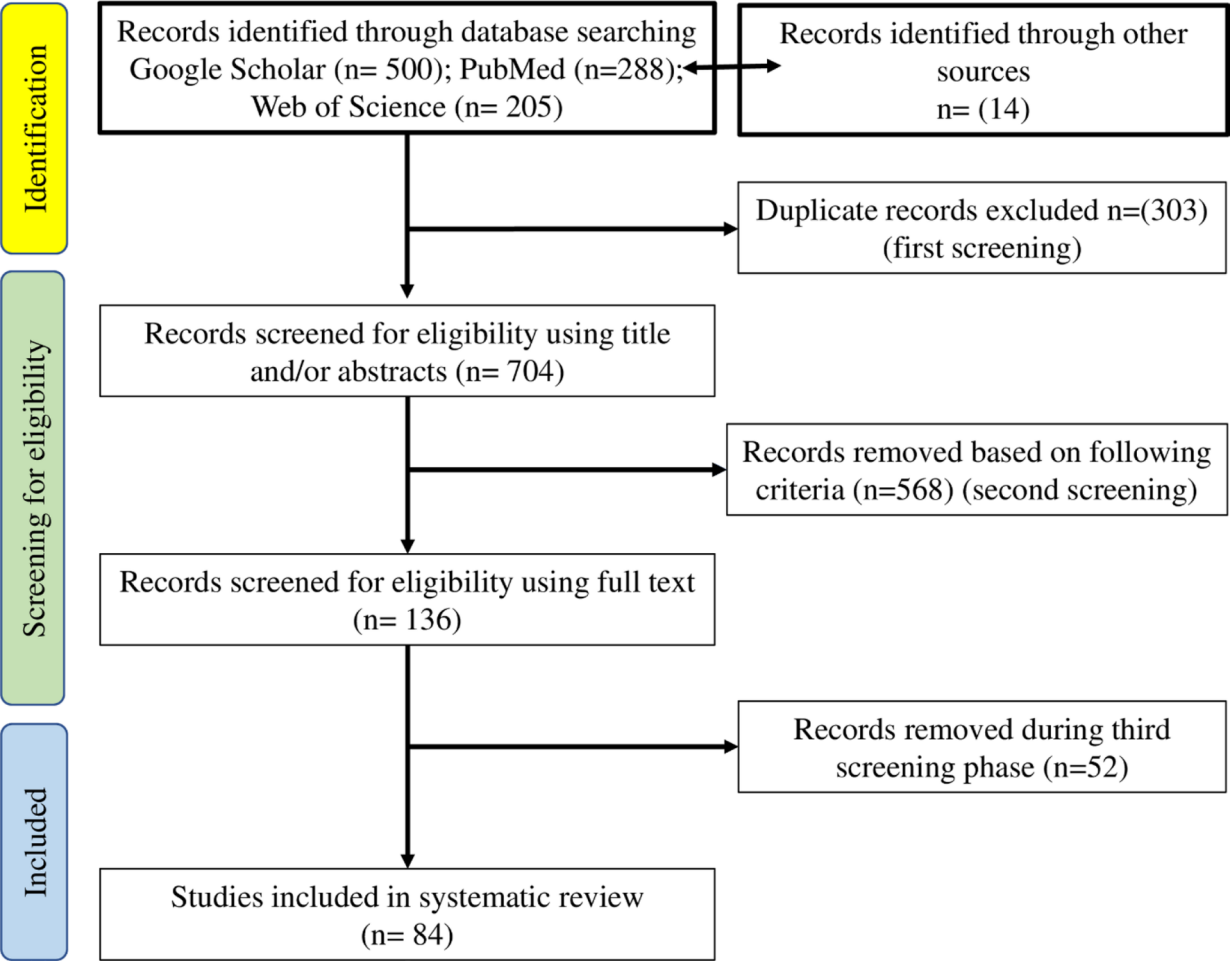
Flow diagram for the literature review

A comprehensive literature search of articles published on the infection of snail intermediate hosts that transmit the trematode parasite in Africa was conducted. The search was conducted using the PubMed, Web of Science and Google Scholar databases from their creation until 24 February 2022 (Fig. 1). The following search terms were used: “(Snails AND Africa) OR (Snails AND diseases)”. The search terms were combined using the Boolean operator “AND/OR”. Our search was limited to peer-reviewed articles published in any language and less than 10 years old. No manual search was done. Relevant articles were also identified from the reference lists of previously identified articles. Zotero v.5 software (www.zotero.org) was used to identify duplicates. We selected articles by analysing their titles and/or abstracts. Only articles that provided (a) data on freshwater snail intermediate hosts of trematodes in different African countries, (b) information on the diagnostic methods used to detect infected snails, and (c) control strategies against snail intermediate hosts were included. Studies without a full text, review articles and meta-analyses were excluded.

## Results and discussion

The results of the search using the Google Scholar, PubMed and Web of Science electronic databases yielded 993 records, and an additional 14 studies were identified and added. After removing duplicates, we examined 704 study titles and/or abstracts and excluded 568 that were not related to the present study and considered irrelevant. Ultimately, 136 full articles were extracted and assessed for eligibility, and 84 were selected for qualitative analysis and included in this review. The article selection process is presented in Fig. 1. Details of the 84 studies are given below.

### Role of freshwater snails in the parasite cycle

FBSPDs are mainly due to trematodes. These snails play an important role as intermediate hosts for several species of trematodes, the best known of which belong to the Schistosomatoidea and Fascioloidea families [6, 12].

**Fig. 2:**
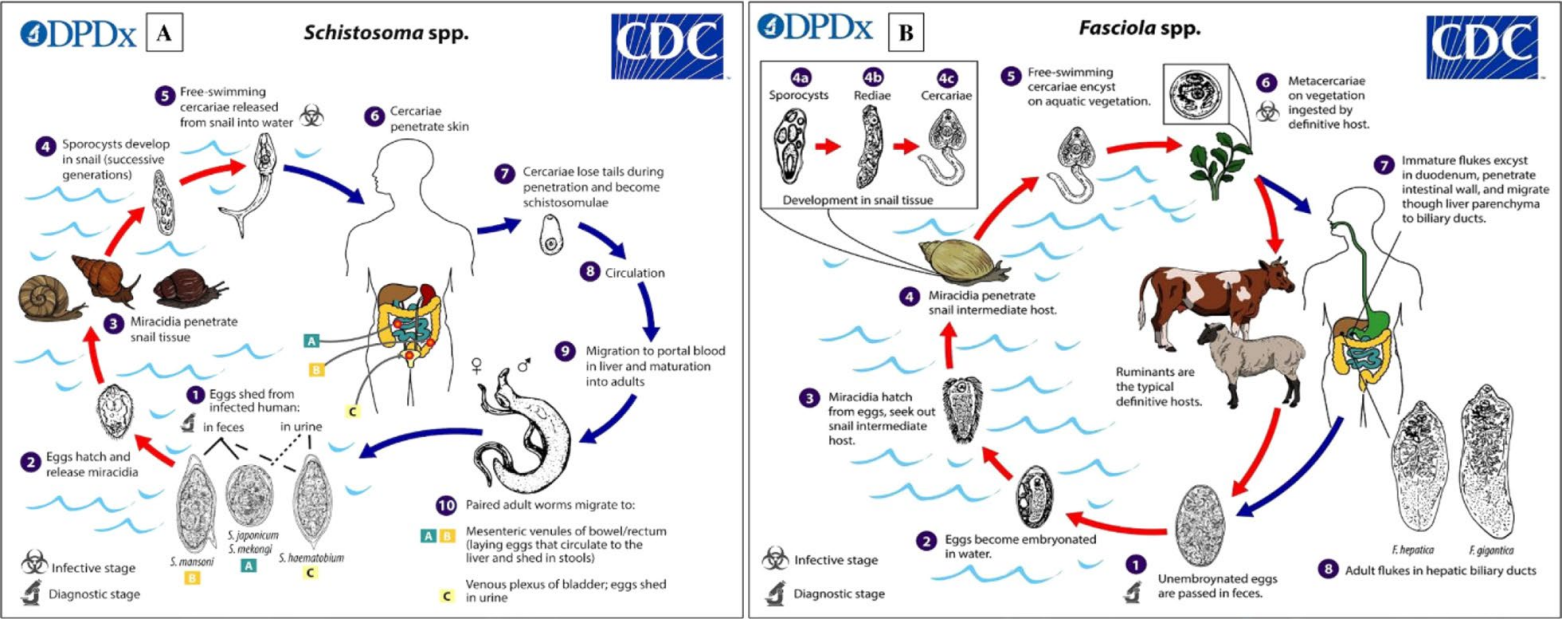
Illustration of the lifecycles of two genera of snail-borne parasites [credit: https://www.cdc.gov/dpdx/schistosomiasis/modules/Schistomes_LifeCycle_lg.jpg and https://www.cdc.gov/dpdx/fascioliasis/modules/Fasciola_LifeCycle_lg.jpg]: **A** Lifecycle of *Schistosoma* spp.: (1) Adult worms reproduce sexually in the venous system of the bladder (S. hæmatobium) or intestine (*S. mansoni*, *S. intercalatum*, S. *guineensis*), producing eggs which are excreted in the urine or faeces. (2) The eggs hatch upon contact with water, releasing miracidia which then enter a specific intermediate snail host. (3) Within the snail host, the miracidia develop into sporocysts and asexually reproduce daughter sporocysts which in turn produce cercariae. (4) The cercariae emerge from the snail and directly penetrate the skin of the human host. (5) After penetrating the skin of the human host, they transform into schistosomules. The schistosomules migrate via the circulatory system to the lungs and then the heart before arriving in the liver where they mature. Once mature, the adult worms emerge from the liver and mate in the mesenteric vessels of the intestine or bladder. **B** Lifecycle of *Fasciola* spp.: (1) immature eggs are evacuated into the bile ducts and passed in the faeces. (2) The eggs embryonate in freshwater for around 2 weeks. (3) The embryonated eggs release miracidia. (4) The miracidia invade a suitable intermediate host, a snail. In the snail, the parasites pass through several stages of development (sporocysts (4a), redia (4b) and cercariae (4c)). (5) The cercariae are released by the snail and encyst as metacercariae on aquatic vegetation or other substrates. (6) Humans and other mammals are infected by ingesting vegetation contaminated with metacercariae (e.g. watercress). (7) After ingestion, metacercariae excyst in the duodenum and penetrate through the intestinal wall into the peritoneal cavity. (8) Immature flukes then migrate through the liver parenchyma to the bile tract, where they become adult flukes and produce eggs. In humans, the maturation of metacercariae into adult flukes generally takes around three to 4 months. The development of *F. gigantica* may take slightly longer than that of *F. hepatica* [92]

These diseases can be divided into groups according to the role of the snail host and the stage of development of the corresponding parasite. The cycle often involves one or two intermediate hosts (IH), or snails are in most cases the first IH. We have classified the diseases into two trematode-related groups. The first is group 1, which corresponds to a direct cycle in which snails are the only intermediate hosts and are infected by miracidia, released by the definitive host (DH) and contaminating the water. This is the case with schistosomes. These miracidia penetrate snails, where asexual reproduction takes place (mother sporocyst and daughter sporocyst stages) leading to the release of thousands of furcocercariae, which swim through the water to infect the definitive host by transcutaneous penetration [15] (Fig. 2A).

This is the case with liver flukes (*Fasciola* spp), the lifecycle of which is characterised by infection of the IH by penetrating miracidium after eggs released by the mammalian DH hatch in the water. The miracidium multiply in the snail into redia and then into cercariae and emerge in the form of mature aquatic larvae (mature cercariae) which will encyst into metacercariae on aquatic plants, which are then ingested by humans or animals (bovids) (Fig. 2B). In humans, the maturation of metacercariae into adult flukes generally takes around three to 4 months. The development of *F. gigantica* may take slightly longer than that of *F. hepatica* [9].

These organisms are divided on the basis of their final habitats in humans: (1) hermaphroditic liver flukes (*Fasciola* spp.), which reside in the bile canal [16] and infect humans when they ingest aquatic plants (e.g. watercress), and (2) bisexual blood flukes (*Schistosoma* spp.), which live in the intestinal or bladder veins (urinary bladder) and infect humans by direct penetration through the skin.

Group 2 corresponds to an indirect cycle involving at least two IHs. One example is echinostomid flukes (Echinostomiasis), which have a life cycle involving a first snail IH (families Planorbidae, Lymnaeidae and Bulinidae) and a second IH including other snails, fish, salamanders and tadpoles. The final host (mammalian: rat, dog, humans, and avian) becomes infected by consuming an infected IH (e.g. snails, clams, fish) [17] Supplementary Fig. S1).

### Importance of asexual reproduction in freshwater snails

Asexual reproduction is an important phase in the multiplication of parasites in the intermediate snail host. The increase in the parasite population by asexual reproduction is based on miracidia, a single one of which can generate hundreds or even thousands of cercariae, which are released into the water by snails under the effect of temperature and light to infect the definitive host when they come into contact with contaminated water. Within snails, miracidia can replicate through several developmental stages. In the case of schistosomes, there are two generations of sporocysts (mother and daughter sporocyst stages) and then cercariae (furcocercariae). In *Fasciola* spp., the development stages are sporocysts, rediae, and then cercariae, which encyst on aquatic vegetation and become metacercariae [18]. Control strategies based on mass drug treatments may be effective in reducing the number of eggs or adult parasites in the human or animal population, but transmission is still maintained by snails carrying the larval stages of these parasites. Thus, controlling the intermediate snail hosts, or the larval stages of parasites within them, has the potential to stop transmission from snails to humans [19].

### Distribution of freshwater snails in Africa

Freshwater snails are intermediate hosts in the lifecycles of various parasites. The geographical distribution of different species of freshwater snails (n = 43) from nine families, and of the infecting parasites, is described in Table 1. *Bulinus* is a group of freshwater snails in the gastropod genus, belonging to the Planorbidae family and the Bulininae subfamily. They are mainly responsible for transmitting larval schistosome parasites that infect humans (notably *S. hæmatobium*), and cattle (*S. bovis* and *S. curassoni*). The distribution of schistosome infections closely follows that of the intermediate host snails *Bulinus* spp., which are endemic in many parts of Africa including Angola, Benin, Chad, Côte d'Ivoire, Egypt, Ethiopia, Gambia, Ghana, Kenya, Niger, Nigeria, Senegal, South Africa, Sudan, Tanzania, and Uganda [20, 28, 29, 31, 39, 42, 44, 45, 48]. The snails found in Africa are *Bu. senegalensis*, *Bu. truncatus*, *Bu. globosus*, *Bu. umbilicatus*, *Bu. forskalii*, *Bu. africanus*, *Bu. crystallinus*, *Bu. angolensis*, *Bu. nasutus* and *Bu. tropicus*. The transmission of schistosomiasis differs significantly from one region to another and depends on the functions performed by the different *Bulinus* species from one ecological region to another. In the region of West Africa, *S. hæmatobium* and *S. bovis* are mainly transmitted by the species *Bu. globosus* and *Bu. truncatus* [4, 21, 25, 31, 55], *Bu. senegalensis* and *Bu. umbilicatus* are also found in Senegal and the Gambia [4, 26] (Table 1). However, in East Africa (Ethiopia, Sudan, Kenya, Tanzania) and southern Africa (Angola, Malawi), in addition to *Bu. globosus* and *Bu. truncatus*, other snail vectors are involved (*Bu. africanus*, *Bu. angolensis* and *Bu. nasutus*) [28, 44].

**Table 1:** List of the distribution of intermediate host snails of trematode species reported in Africa, based on studies conducted between 2012 and 2022

Freshwater snail families, sub-families and species	Geographical distribution	Trematode parasites detected in snails or in free larval form (cercariae)*															References
		*Sh*	*Sb*	*Sc*	*Sm*	*Sr*	*Fh*	*Fg*	*Xs*	*Gc*	*Ac*	*Scs*	*ASc*	*Ec*	*Sc*	*Pc*	
Bulinidae/Bulininae																	
* Bu. senegalensis*	Gambia, Senegal, Kenya	( +)	( +)	−	−	−	−	−	−	−	−	−	−	−	−	−	[4, 20, 21]
* Bu. forskalii*	Angola, Benin, Chad, Côte d’Ivoire, Ethiopia, Gambia, Mali, Niger, Nigeria, Senegal, Tanzania, Uganda	−	( +)	−	−	−	−	−	( +)	−	−	( +)	−	−	−	−	[22–30]
* Bu. umbilicatus*	Senegal	( +)	( +)	( +)	−	−	−	−	−	−	−	−	−	−	−	−	[4, 31]
* Bu. globosus*	Angola, Benin, Côte d'Ivoire, Ethiopia, Kenya, Mali, Niger, Nigeria, Senegal, Tanzania	( +)	( +)	−	−	−	−	−	( +)	−	( +)	( +)	( +)	( +)	( +)	−	[22, 24, 25, 28, 31–36]
* Bu. truncatus*	Benin, Cameroon, Chad, Côte d'Ivoire, Egypt, Gambia, Ghana, Kenya, Niger, Nigeria, Senegal, Sudan, Uganda	( +)	( +)	−	−	−	−	−	( +)	−	( +)	( +)	−	( +)	−	−	[20, 24–31, 36–40]
* Bu. tropicus*	South Africa, Tanzania, Uganda	−	−	−	−	−	−	−	−	−	−	( +)	−	−	( +)	−	[30, 41]
* Bu. nasutus*	Kenya, Tanzania	( +)	−	−	−	−	−	−	−	−	−	( +)	−	−	−	−	[32–35]
* Bu. africanus*	Malawi, Kenya, Tanzania	( +)	−	−	−	−	−	−	−	( +)	−	( +)	−	−	−	−	[42–44]
* Bu. ugandae*	Kenya	−	−	−	−	−	−	−	( +)	−	−	−	−	−	−	−	[45]
* Bu. angolensis*	Malawi	( +)	−	−	−	−	−	−	−	−	−	−	−	−	−	−	[44]
* Bu. crystallinus*	Angola	−	−	−	−	−	−	−	−	−	−	−	( +)	( +)	−	−	[46]
*Cleopatra bulimoides*	Egypt, Senegal, Sudan	−	−	−	−	−	−	−	−	−	−	−	−	−	−	−	[16, 21, 38, 39]
*C. colbeaui*	Nigeria	−	−	−	−	−	−	−	−	−	−	−	−	−	−	−	[23]
Planorbidae/Planobinae																	
* Biomphalaria pfeifferi*	Benin, Cameroon, Chad, Côte d’Ivoire, Ethiopia, Ghana, Kenya, Mali, Niger, Senegal, Sudan, Tanzania	−	−	−	( +)	−	−	−	( +)	−	( +)	-	( +)	( +)	( +)	−	[20, 22, 24, 25, 27, 29, 31, 38–40, 42, 43, 45, 47–50]
* Bi. choanomphala*	Kenya, Uganda	−	−	−	( +)	( +)	−	−	−	−	−	( +)	−	−	−	−	[47, 49, 51]
* Bi. sudanica*	Ethiopia, Kenya, Uganda	−	−	−	( +)	−	−	−	−	−	−	( +)	−	( +)	−	−	[47–49]
* Bi. stanleyi*	Uganda	−	−	−	( +)	−	−	−	−	−	−	( +)	−	−	−	−	[47, 49]
* Bi. alexandrina*	Egypt	−	−	−	( +)	−	−	−	−	−	−	( +)	−	−	−	−	[37, 52]
* Gyraulus spp*	Gambia	−	−	−	−	−	−	−	−	−	−	−	−	−	−	−	[26]
* Gyraulus costulatus*	Angola	−	−	−	−	−	−	−	−	−	−	−	−	−	−	−	[46]
Lymnaeidae																	
* Lymnaea truncatula*	Egypt, Ethiopia, Lesotho, Morocco, Tanzania, Uganda	−	−	−	−	−	( +)	−	−	−	−	−	−	−	−	−	[13, 48, 53]
* Lymnaea natalensis*	Angola, Benin, Ethiopia, Kenya, Mali, Niger, Nigeria, Senegal, South Africa, Uganda, Tanzania	−	−	−	−	−	−	( +)	( +)	( +)	−	−	( +)	( +)	( +)	−	[22, 23, 25, 41–43, 45, 46, 48, 54]
* Pseudosuccinea columella*	Egypt	−	−	−	−	−	−	( +)	−	−	−	−	−	( +)	−	−	[16]
Viviparidae																	
* Bellamya constricta*	Tanzania	−	−	−	−	−	−	−	−	−	−	−	−	−	−	−	[42]
* Be. unicolor*	Mali, Nigeria, Senegal	−	−	−	−	−	−	−	( +)	−	−	−	−	−	−	−	[21–23, 45]
Thiaridae																	
* Melanoides tuberculata*	Angola, Benin, Côte d'Ivoire, Egypt, Nigeria, Senegal, Sudan, Tanzania	−	−	−	−	−	−	−	( +)	−	−	−	−	−	−	−	[21, 23, 25, 38, 39, 42, 45, 46, 55]
Ampullaridae																	
* Pila ovata*	Kenya, Sudan, Uganda	−	−	−	−	−	−	−	( +)	−	−	−	−	( +)	−	( +)	[38, 39, 45, 56]
* Pila werneri*	Nigeria	−	−	−	−	−	−	−	−	−	−	−	−	−	−	−	[23]
* Pila acuta*	Côte d'Ivoire	−	−	−	−	−	−	−	−	−	−	−	−	−	−	−	[24]
* Lanistes spp*	Gambia	−	−	−	−	−	−	−	−	−	−	−	−	−	−	−	[26]
* Lanistes lybicus*	Nigeria	−	−	−	−	−	−	−	−	−	−	−	−	−	−	−	[23]
* Lanistes varicus*	Mali, Nigeria	−	−	−	−	−	−	−	−	−	−	−	−	−	−	−	[22, 23]
* Lanistes ovum*	Angola	−	−	−	−	−	−	−	−	−	−	−	−	−	−	−	[46]
* Potadoma moerchi*	Kenya	−	−	−	−	−	−	−	−	−	−	−	−	−	−	( +)	[45]
Physidae																	
* Physa* sp*.*	Nigeria, Côte d'Ivoire	−	−	−	−	−	−	−	−	−	−	−	−	−	−	−	[36, 55]
* Physa marmorata*	Benin	−	−	−	−	−	−	−	−	−	−	−	−	−	−	−	[57]
* P. heterostropha*	Egypt	−	−	−	−	−	−	−	−	−	−	−	−	−	−	−	[16]
* Physa acuta*	Angola, Côte d'Ivoire, Sudan	−	−	−	−	−	−	−	−	−	−	−	−	−	−	−	[38, 39, 46]
Succinidae																	
* Succinea *sp.	Angola, Egypt	−	−	−	−	−	−	−	−	−	−	−	−	−	−	−	[16, 46]
Neritidae																	
* Theodoxus anatolicus*	Egypt	−	−	−	−	−	−	−	−	−	−	−	−	−	−	−	[16]
Bithyniidae																	
* Gabbiella humerosa*	Nigeria, Uganda	−	−	−	−	−	−	−	−	−	−	−	−	−	−	−	[23, 56]

^*^Sh Schistosoma hæmatobium, Sb S. bovis, Sc S. curassoni, Sm S. mansoni, Sr S. rodhaini, Fh Fasciola hepatica, Fh Fasciola gigantica, Xs Xiphidiocercariae, Gc Gymnocephalous cercariae, Ac Amphistoma cercariae, Scs Schistosoma spp*. cercariae, ASc Avian Schistosoma cercariae, Ec Echinostoma cercariae, Sc Strigea cercariae, Pc Parapleurolophocercous cercaria*

( +) = Presence of infection; (− ) = No information available

*Biomphalaria* belongs to the genus of freshwater gastropod snails, which are part of the family Planorbidae. They are the main intermediate hosts for the transmission of *S. mansoni* infection leading to intestinal schistosomiasis and are generally found in tropical freshwater ponds in sub-Saharan Africa. *Biomphalaria* species cannot survive outside freshwater, unlike *Bulinus* which can survive in temporary pools. There are several species of *Biomphalaria* that are known vectors for the transmission of intestinal schistosomiasis in Africa. In this review, five of these were highlighted, namely *Bi. pfeifferi*, *Bi. alexandrina*, *Bi. choanomphala*, *Bi. stanleyi* and *Bi. sudanica* [37, 47–49, 58]. Other Planorbidae have been reported in Angola (*Gyraulus costulatus*) and The Gambia (*Gyraulus* sp.) [26, 46]. Studies have shown that *Biomphalaria* spp. can reside in slow moving waters with little wave action [59]. This seems to be a favourable condition for miracidia to infest snails and undergo asexual reproduction to form cercariae. Several species of *Biomphalaria* are found in the Horn of Africa, in places such as Lake Victoria in Uganda, where significant transmission occurs [49], as well as in Kenya, Tanzania and Ethiopia [47, 48]. However, the predominant species in West Africa remains *Bi. pfeifferi*, which is strongly implicated in the transmission of *S. mansoni*. *Bi. alexandrina* is widely distributed in Egypt [37, 52].

The studies examined show that *Lymnaea truncatula* and *L. natalensis* coexist in certain East African countries, notably Ethiopia, Tanzania and Uganda, where *F. hepatica* and *F. gigantica* have been documented [42, 43, 48]. However, in other countries such as Egypt (East Africa), Nigeria, Niger, Senegal, Benin (West Africa), Angola and South Africa (southern Africa), *L. natalensis* has been reported as the IH of *F. gigantica* [23, 25, 41–43, 45, 46, 48, 54]*.* We have documented the *Pseudosuccinea columella* species only in Egypt, where it is found as an intermediate host of *F. gigantica* [16].

Other snail families have been documented in Africa, notably Thiaridae, Ampullaridae, Physidae, Succinidae, Neritidae and Bithyniidae [16, 23, 46]. The species in these families are usually vectors or sometimes hosts of certain trematodes of veterinary interest, in particular *Echinostoma* cercariae or Xiphidiocercariae, as is the case with the Ampullaridae [38, 39, 45, 56]. The latter are also known to be bio-agents that predate other snail intermediate hosts.

### Examples of snail-borne parasitic diseases in Africa

#### Schistosomiasis

Schistosomiasis is caused by worms belonging to the genus *Schistosoma*, which infect the mammalian host by transcutaneous penetration. It is a water-borne disease, involving different species of schistosomes. These species have a very broad parasite spectrum worldwide, particularly in subtropical Africa, with a geographical distribution that follows that of their hosts. These species have a very broad parasite spectrum worldwide, particularly in subtropical Africa, with a geographical distribution that follows that of their hosts. Schistosomiasis is one of the 20 neglected tropical diseases currently listed by the WHO [1], and represents a parasitic disease of considerable medical and veterinary importance in tropical and sub-tropical regions, especially in SSA [60].

#### Epidemiology and distribution

Schistosomiasis is one of the most widespread parasitic diseases in the world, with confirmed transmission in 78 countries [3]. In 2021, it was estimated that at least 251.4 million people needed preventive treatment against schistosomiasis in low-and middle-income countries in tropical regions [3]. In 2021, according to the WHO, schistosomiasis is now largely restricted to SSA, in poor communities without access to safe drinking water and adequate sanitation, where 90% of cases occur [3]. In this region, 600 million people are at risk of urogenital schistosomiasis infection [3]. In total, six species (*Schistosoma hæmatobium*, *S. mansoni*, *S. japonicum*, *S. mekongi*, *S. guineensis* and *S. intercalatum*) are responsible for the two major forms of the disease. *S. mansoni*, *S. japonicum*, *S. mekongi*, *S. guineensis* and S*. intercalatum* cause intestinal schistosomiasis and *S. hæmatobium* causes urogenital form. However, only four species are present in Africa, namely *S. hæmatobium*, *S. intercalatum*, *S. guineensis* and *S. mansoni*. *S. mansoni* is transmitted by snails of the genus *Biomphalaria*, the most endemic of which in Africa is the *Bi. pfeifferi* species [27, 29, 31, 38, 40, 47, 48]. In contrast, *S. hæmatobium*, which causes human urogenital schistosomias is the most widespread species [3], and is transmitted by Bulininae snails, mainly species in the genus *Bulinus* (Supplementary Fig. S2).

*S. mansoni* intestinal schistosomiasis is the most common form of schistosomiasis in the world, with a geographical distribution in Africa which closely follows that of *S. hæmatobium* (Supplementary Fig. S2). In addition to *S. intercalatum* and *S. guineensis*, *S. mansoni* causes intestinal and hepatic and intestinal schistosomiasis in mammals [3]. Tanzania has the second highest burden of schistosomiasis in the region after Nigeria [61, 62]. In this review, *S. rodhaini*, a parasite mainly affecting rodents, was found in *Bi. choanomphala* in Uganda [63].

**Fig. 3:**
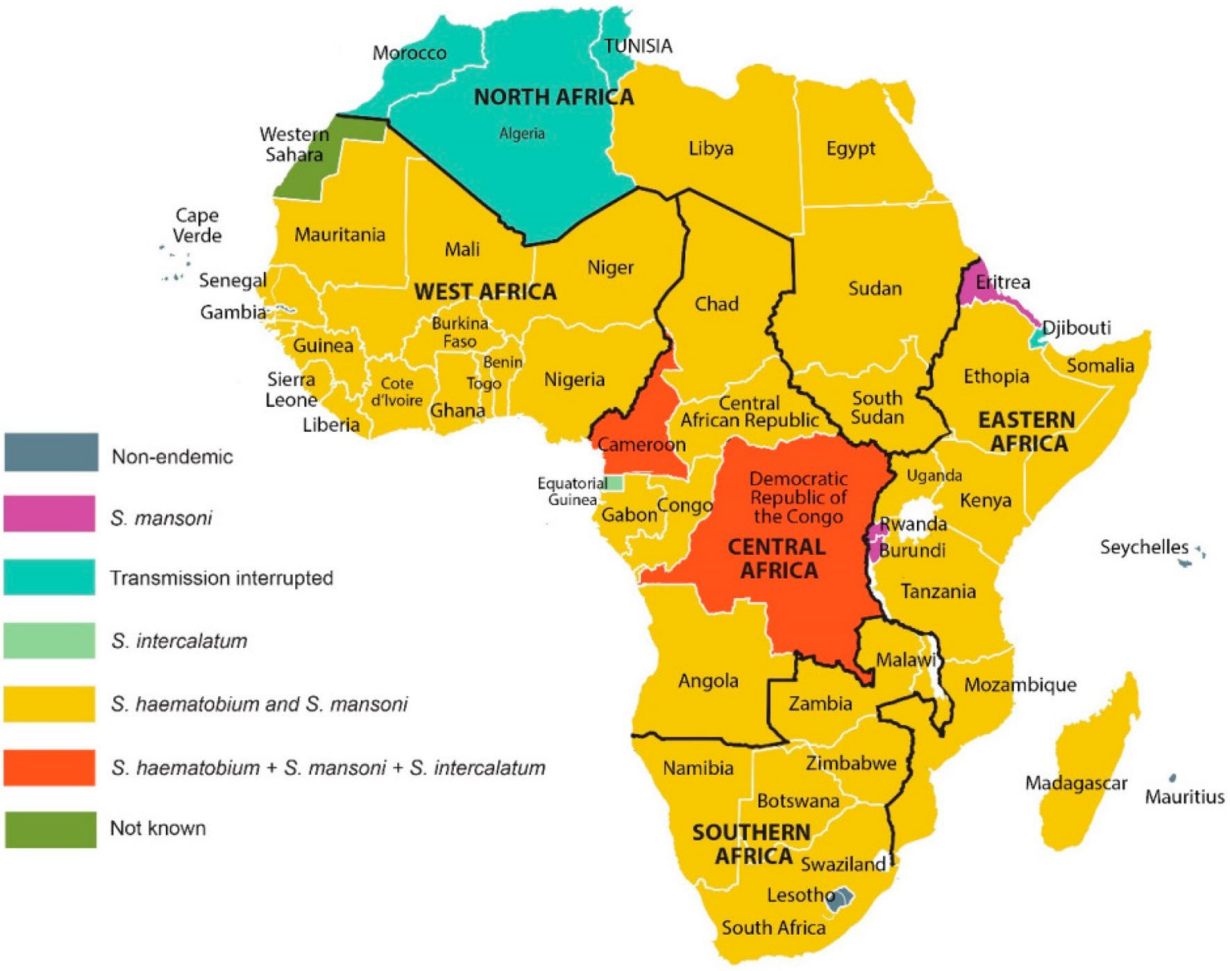
Distribution of schistosome species in Africa [93]

The urinary form, due to *S. hæmatobium*, is present in most countries on the African continent and in Madagascar [3]. *S. intercalatum* and *S. guineensis*, two closely related species, are found in tropical rainforest areas in central Africa [42]. In addition to these species, other schistosomes exclusive to cattle are found throughout the continent. Some of these species are genetically related and form the *S. hæmatobium* complex, grouping together all the human and animal species related to *S. hæmatobium* and widely distributed in Africa (Fig. 3).

### The *Schistosoma hæmatobium* complex

Members of the *S. hæmatobium* complex include three species that are pathogenic to humans (*S. hæmatobium*, *S. intercalatum* and *S. guineensis*) and five others that infect animals, especially wild and domestic ruminants (*S. bovis*, *S. curassoni*, *S. mattheei*, *S. leiperi* and *S. margrebowiei*). Neither *S. leiperi* nor *S. margrebowiei* are widely distributed in Africa. These two species have been noted in East Africa but have not been the subject of in-depth research [64]. Consequently, the group as a whole is of immense medical and veterinary importance. The species that make up the group are related and can interact and possibly hybridise. This hybridisation may result in strains with a broader host spectrum and/or strains which are more resistant to treatment [31].

### Fascioliasis

Fascioliasis is a zoonotic trematode disease transmitted by snails, which is of major health and economic importance [65]. Fascioliasis affects both domestic ruminants and humans. In humans, the disease is characterised by the destruction of liver tissue and the bile tract. This provokes inflammatory responses leading to hepatomegaly or cirrhotic liver, accompanied by diarrhoea and anaemia. *Lymnaea* snails are suitable intermediate hosts for *Fasciola* spp. and live in contact with the definitive host (humans or cattle) around riverbanks [65]. We have two main species, namely *F. hepatica* Linne, 1758 and *F. gigantica* Cobbold, 1855.

### Epidemiology and distribution

**Fig. 4:**
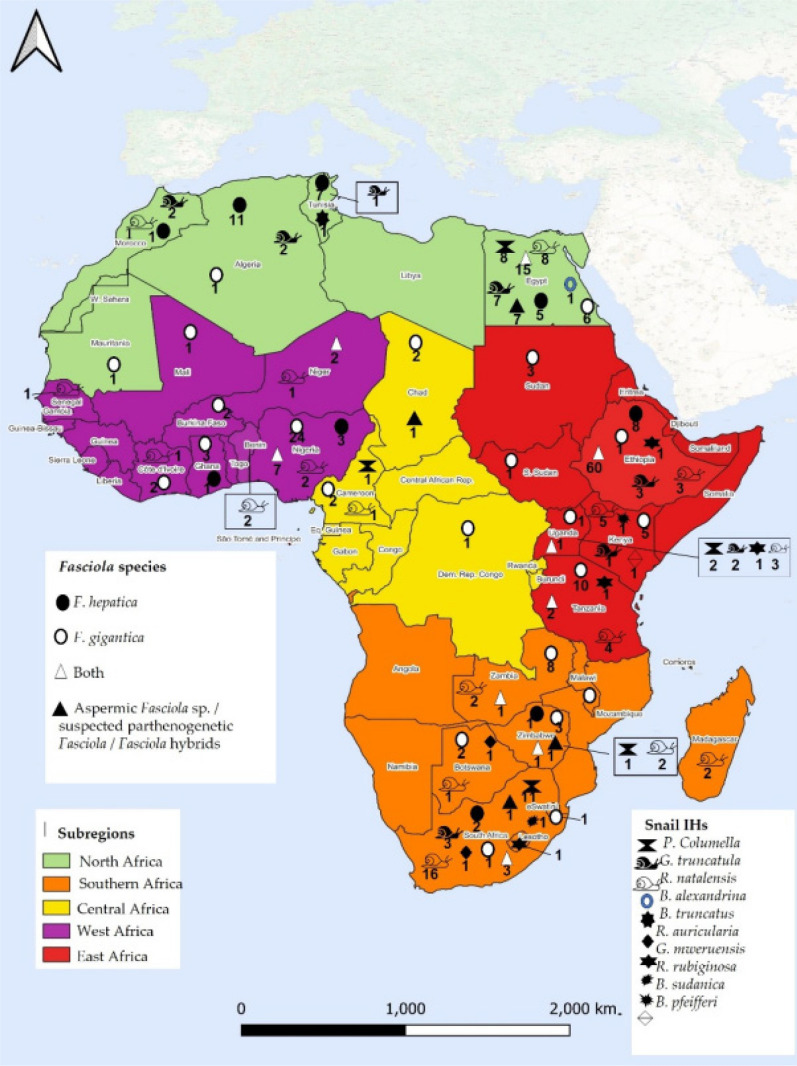
A map showing the geographical distribution and occurrence of *Fasciola* spp. and their intermediate hosts, snails, in Africa. The taxa reported are symbolised next to the number of studies in each country [94]

Fascioliasis is one of the most significant liver diseases of herbivores. It is caused by *Fasciola* spp. infection. Fascioliasis is thought to cause economic losses in addition to human suffering [53]. *F. hepatica* has a wider distribution than its tropical counterpart, *F. gigantica*, but their geographical distribution overlaps in many African countries, particularly Egypt, where both species are present [16, 54]. In Egypt, however, the transmission of *F. gigantica* involves another species of snail in the genus *Pseudosuccinea*. Grabner et al. [16] highlighted the ability of *P. columella* to transmit *F. gigantica* as well as its abundance as an invader in irrigation canals in the Fayoum governorate in Egypt, with a prevalence of 3.83%. The endemicity of human fascioliasis has been noted in the North Africa, particularly in Egypt [16], and Ethiopia [66]. Human fascioliasis is also present in other African countries such as Chad [67], South Africa [68] and Zimbabwe [69]. However, it remains less studied in less developed countries [70]. Animal fascioliasis has been widely reported in almost all the countries in the African equatorial belt [66], and in eastern and southern Africa [53] (Fig. 4).

In addition to the widespread geographical distribution of the FSBPDs mentioned above, other FSBPDs have been recorded in Africa. These include paramphistomiasis, a disease of domestic and wild ruminants caused by Paramphistomoidea Fischoeder, 1901. This is a food-borne disease caused by trematodes belonging to several genera, including *Calicophoron*, *Cotylophoron*, *Explanatum*, *Gigantocotyle* and *Paramphistomum* [71]. The larval forms of these parasites encyst as metacercariae in semi-aquatic plants, which are then consumed by DHs (mammals, especially domestic ruminants). The *Paramphistoma* cycle is similar to that of the large liver fluke, *Fasciola* spp. These two parasites share a common host in *Lymnaea* spp. The main difference between the two diseases lies in the location of the parasites in the animals’ bodies, and their development. In temperate regions, paramphistomosis has a moderate impact on livestock, whereas it causes greater losses in African countries due to the poor general condition of ruminants [72]. A study carried out on snails from Kenya, Tanzania and Egypt revealed *Paramphistoma* cercariae in *Bu. forskalii* (*Calicophoron* spp. and *C. microbothrium*), *Bi. sudanica* (Paramphistomoidae), *Ceratophallus natalensis* (Gastrothylacidae), and *Gyraulus euphraticus* [73]. Ismail et al. (2022) [38] identified *Amphistoma* cercariae in *Bu. truncatus* and *Bi. pfeifferi* as the first intermediate host.

Echinostomiasis is also a food-borne trematodiasis, caused by trematodes of the family Echinostomatidae Looss, 1899. Echinostomid flukes have a multi-host (indirect) life cycle involving a first IH snail and a second IH including other snails, bivalves, fish, salamanders and tadpoles [74]. The final host (birds, carnivores, rodents and humans) becomes infected through consumption of metacercariae from the infected IH. Incidence is highest in areas where freshwater snails, clams, raw or undercooked fish and amphibians are consumed. A study by Laidemitt et al. [74] highlighted the diversity of echinostomes transmitted in Africa, focusing on the larval forms present in the IH *Bulinus* spp. and *Biomphalaria* spp. Similarly, *Echinostoma* cercariae have been found in *Pila ovata* (Ampullariidae) [45]. Mereta et al. [48] found a 36% prevalence of infection of *Bi. pfeifferi* by *Echinostoma* cercariae in the Omo Gibe River basin in south-west Ethiopia.

### Detection of parasitic infections in snails

Several methods are used to detect parasites in intermediate snail hosts. Table 2 presents the information extracted from seven articles resulting from bibliographical research and developing some methods of parasitic diagnosis in snails.

**Table 2:** Methods for detecting parasites in snails

Country	Freshwater snail species	Identification technique(s)	Advantages	Limitations	Ref
Tanzania	*Bulinus globosus* and *Bu. nasutus*	Conventional and nucleic-acid amplification diagnostics (cercarial shedding and real-time PCR DraI)	The PCR approach makes it possible to detect pre-patent infections, unlike cercarial excretion, which shows infection only when the cercariae have matured	Although PCR detects pre-patent infections, these often do not develop to patency. This may be due to host-parasite incompatibility and/or snail longevity. This PCR is non-specific and detects a group of parasites	[76]
Sudan	*Bi. pfeifferi*, *Bu. truncatus, Bu. forskalli, Cleopatra bulimoides, M. tuberculata, Physa acuta*, and* Lymnaea natalensis*	Conventional diagnostics (cercarial shedding test)	The excretion of cercariae and the use of morphological characteristics to identify associated trematodes are less costly than the use of molecular techniques, but more suitable for use in the field. They can also be used to assess a snail’s ability to transmit a parasite	The use of cercarial excretion and morphological characteristics to identify snails and the trematodes associated with them is not precise and is often limited to the genus, unlike more reliable molecular techniques	[58]
Tchad	*Bu. truncatus*, *Bu. forskalii*, and *Bi. pfeifferi*				[27]
Ethiopia	*Bi. pfeifferi, Bi. sudanica, B. globosus, Bu. forskalii*, and *Lymnaea natalensis*				[48]
Egypt	*Bi. alexandrina*	Conventional and nucleic-acid amplification diagnostic methods (cercarial shedding and snail crushing) and PCR	PCR had an average 100% sensitivity and specificity. PCR techniques could detect preclinical or latent infection	The cost of reagents needed for PCR is relatively high when compared to conventional methods. PCR methods require skilled and trained technicians	[77]
Tanzania	*Biomphalaria* sp	Nucleic-acid amplification diagnostics (real-time PCR)	More sensitive for detecting infections than cercarial excretion. Faster and allows better assessment of infection rates	The technique is expensive and cannot be used directly in field settings for routine assessment of infection in snails. Further testing is required to rule out cross-reactions with other trematodes	[78]
Egypt	*Bu. truncatus* and *Bi. alexandrina*	Conventional and nucleic-acid amplification diagnostic (microscopic techniques (cercarial shedding, snail crushing) and PCR)	PCR is more sensitive for detecting infections than conventional techniques. Conventional techniques only detect parasites when they are mature. PCR detects them earlier and is a potential tool that can be used to monitor schistosome transmission on a large scale. However, there are disadvantages of using PCR in the field	The results obtained when conventional techniques are used to detect infection are underestimated because they only detect the mature stages of the parasite. Although PCR has a higher sensitivity than conventional techniques, there are cases of inaccurate detection	[37]

### Microscopic methods

As a general rule, infections in snails are detected by direct observation (microscopy). Snails are examined for sporocysts by crushing, or they are kept alive until cercariae are shed after exposure to light (Shedding test). The cercariae are then observed directly under the microscope [29, 37, 58].

#### Snail crushing

The technique involves using a microscope to look for the development of cercariae and sporocysts. Sporocysts, which are often located inside the snail’s foot and mantle, cannot be observed with the naked eye. Observation involves destroying the shell, dissecting the viscera and examining them under a binocular magnifying glass or microscope to collect immature parasites. This technique has been used by many authors to monitor the intensity of natural snail infestations [54]. This technique is inaccurate in the early prepatent stage and is not ideal for large-scale field snail screening.

#### Cercarial shedding test

Patent infections in snails are defined by the excretion of cercariae, hence their ability to transmit parasites. Snails are generally tested for cercarial emergence immediately after collection or several days after collection.

**Fig. 5:**
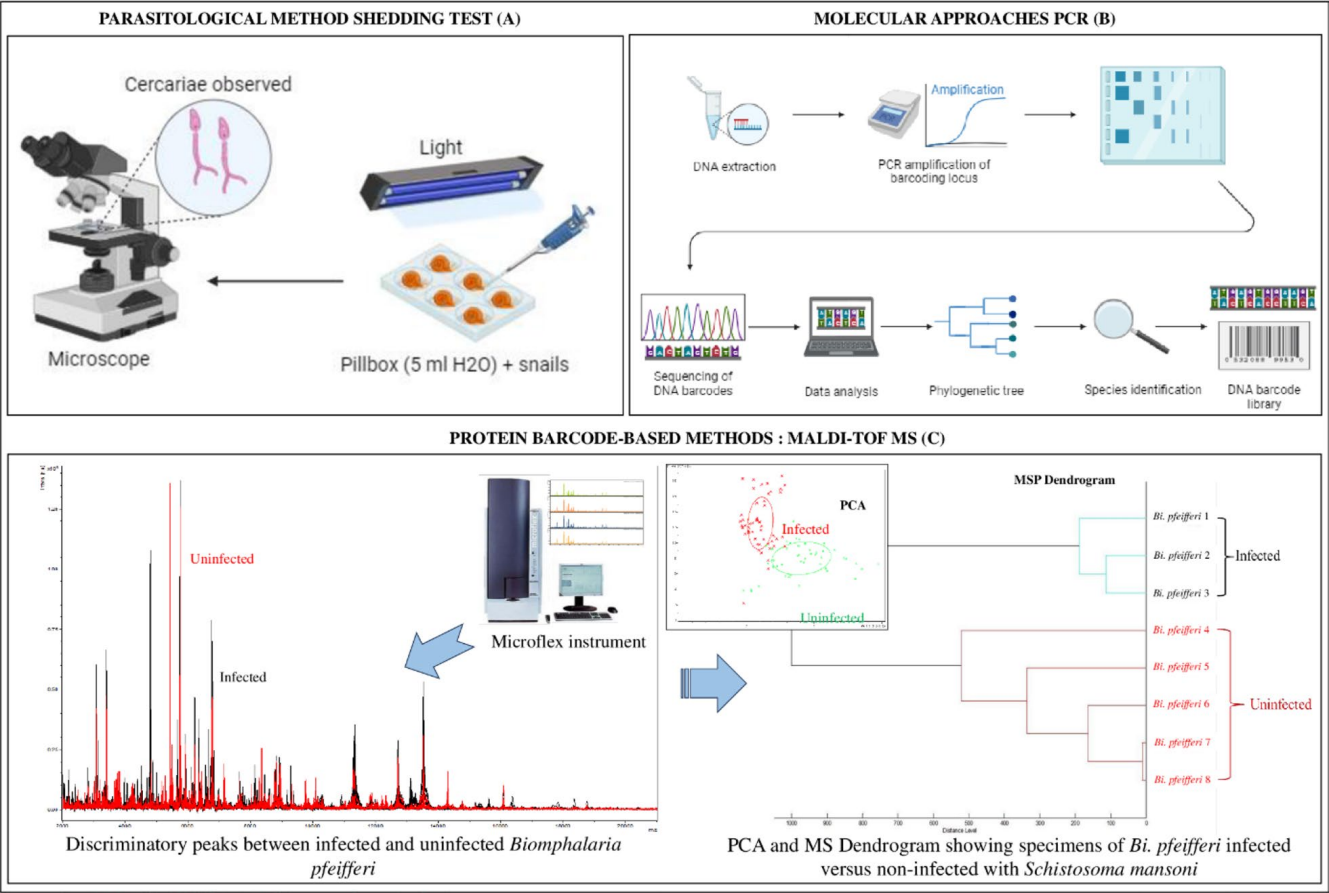
Methods for detecting parasitic infections in snails: **A** cercarial shedding test, **B** PCR and sequencing, and **C** MALDI-TOF MS

Studies have used cercarial excretion tests to assess the infectious profile of snails [29, 58, 75]. This involves exposing living snails to natural or artificial light to stimulate the emission of larval forms of the parasites. The cercariae were observed directly under a microscope or stained with iodine and Ehrlich’s haematoxylin or Nile blue sulphate [45] in order to better observe morphological characteristics, and were then identified to subtype, genus and species level using morphological criteria. These criteria included snail excreting cercarial species, cercarial swimming behaviour, and resting position (Fig. 5A). Mohammed et al. [58], determined that 14.1% of snails excreted different types of cercariae including *Schistosoma*, *Amphistoma*, *Echinostoma*, *Xiphidiocercariae* and *Parapleurolophocercariae.*

Shedding tests have a number of limitations and are not suitable for large-scale field studies. To improve test accuracy, it is often necessary to hold snails that have not shed for long periods of examination in the laboratory. However, mortality induced by observation time can be high and makes it difficult to perform large-scale infection tests on field snails. However, the sensitivity and/or specificity of these classic microscopic techniques (cercarial crushing and shedding tests) is problematic, hence the need for highly sensitive and specific techniques to detect the parasite in snails, such as the molecular PCR approach.

### DNA-based methods

#### Conventional PCR and real-time polymerase chain reaction (RT-PCR)

The classic PCR-based molecular technique is sensitive and specific for the detection of parasites in intermediate snail hosts [77]. It can be performed directly on DNA extracted from the snail or from cercariae previously isolated on Whatman FTA Classic cards [37, 51, 79].

The most commonly used approach for detecting infections in snails involves RT-PCR amplification of repeated sequences, including Dra1 (*S. hæmatobium* complex) or SM1-7 (*S. mansoni* group). Fuss et al. [78] detected a 35.4% infection rate of S. mansoni in *Biomphalaria* spp. in Tanzania using RT-PCR targeting a 121 bp tandem repeat sequence of *S. mansoni* strain SM 1–7 with Sm primers [78]. A study by Gaye et al. [4] using Dra1 RT-PCR detected 29.1% infection with the *S. hæmatobium* group in *Bulinus* spp. collected in Senegal. Allan et al. (2013) [76] in Zanzibar compared the infection rate of *Bu. globosus* using conventional diagnosis and real-time PCR. An infection rate of 3.96% was observed from conventional cercarial excretion diagnosis, while a higher infection rate of 40–100% was detected with RT-PCR diagnosis. However, specificity may be problematic with Dra1 RT-PCR for *S. haematobium* complex.

To confirm RT-PCR results, it is necessary to perform classical PCR or specific amplicon sequencing (Fig. 5B). Some of the works included in this review have used classical PCR to characterize schistosome species. This consists in amplifying gene regions specific to the parasite species. Gaye et al. [4] used the mitochondrial COI gene as a DNA barcode to detect and discriminate *S. hæmatobium* and S. bovis from *Bulinus* snail DNA extracts. In addition, Aboelhadid et al. [37] used conventional PCR and conventional diagnostics to compare their ability to detect S. mansoni infection in snails. The PCR test detected schistosomes in snails in contrast to conventional diagnostic methods [77].

#### Loop-mediated amplification (LAMP)

In some cases, detection methods have evolved from PCR amplification to loop-mediated isothermal amplification (LAMP). This is based on a DNA amplification reaction at the same temperature. LAMP appears to be an interesting new technique for detecting trematodes in the intermediate host [80]. It uses four specially developed primers to recognise six different sequences on the target gene. According to [80], this technique has the advantage of reducing costly equipment and being easily adaptable to field laboratories. This is all the more true in Kenya, where local survey teams with no experience of molecular biology acquired operational expertise in the LAMP technique in the space of a few hours. In this study, the authors were able to detect comparable infection rates of *Bulinus* spp. by *Schistosoma* spp. between the LAMP technique (48.5%) and qPCR (52.4%) [80]. These results show the potential of the LAMP test for DNA amplification in conditions where large-scale molecular biology equipment is unavailable, particularly in field laboratories.

#### Antibody-based methods

An immunological technique has been developed to assess trematode infections in snail, using monoclonal antibodies [81]. The ELISA technique gave promising results, but is relatively cumbersome to set up, insofar as substantial equipment (spectrophotometer, oven) and complex biological reagents (monoclonal antibodies) are required.

### Protein barcode-based methods

#### MALDI-TOF MS

Matrix-assisted laser desorption/ionisation time-of-flight mass spectrometry (MALDI-TOF MS) is a protein tool used routinely in clinical microbiology. More recently, it has been presented as an alternative tool for the rapid identification of freshwater snail intermediate hosts of schistosomes [21, 82, 83]. Studies have also shown that MALDI-TOF is a reliable technique for high-throughput identification of *Schistosoma* cercariae of medical and veterinary importance and could be useful for field surveys in endemic areas [84]. There is currently no published work on the detection of pre-patent infection in snails by MALDI-TOF MS. However, we have obtained preliminary results on the identification of *Schistosoma* spp. infected versus uninfected *Bi. pfeifferi* using the MALDI-TOF MS protein tool (Gaye et al. in press). In this study, the two groups were distinguished using spectral profiles showing discriminative peaks, the dendrogram and Principal Component Analysis (Fig. 5C).

#### Control of intermediate host snails

Despite the general acceptance of the use of chemotherapeutic drugs in the treatment of major trematodiasis, control of intermediate snail hosts plays an important role in FSBD control strategies. The use of molluscicides, complementing control efforts based on chemotherapy, sanitation and public health education, offers considerable potential for reducing disease transmission. Molluscicides can be chemical (Niclosamide) or natural (plant extracts) [85].

**Fig. 6:**
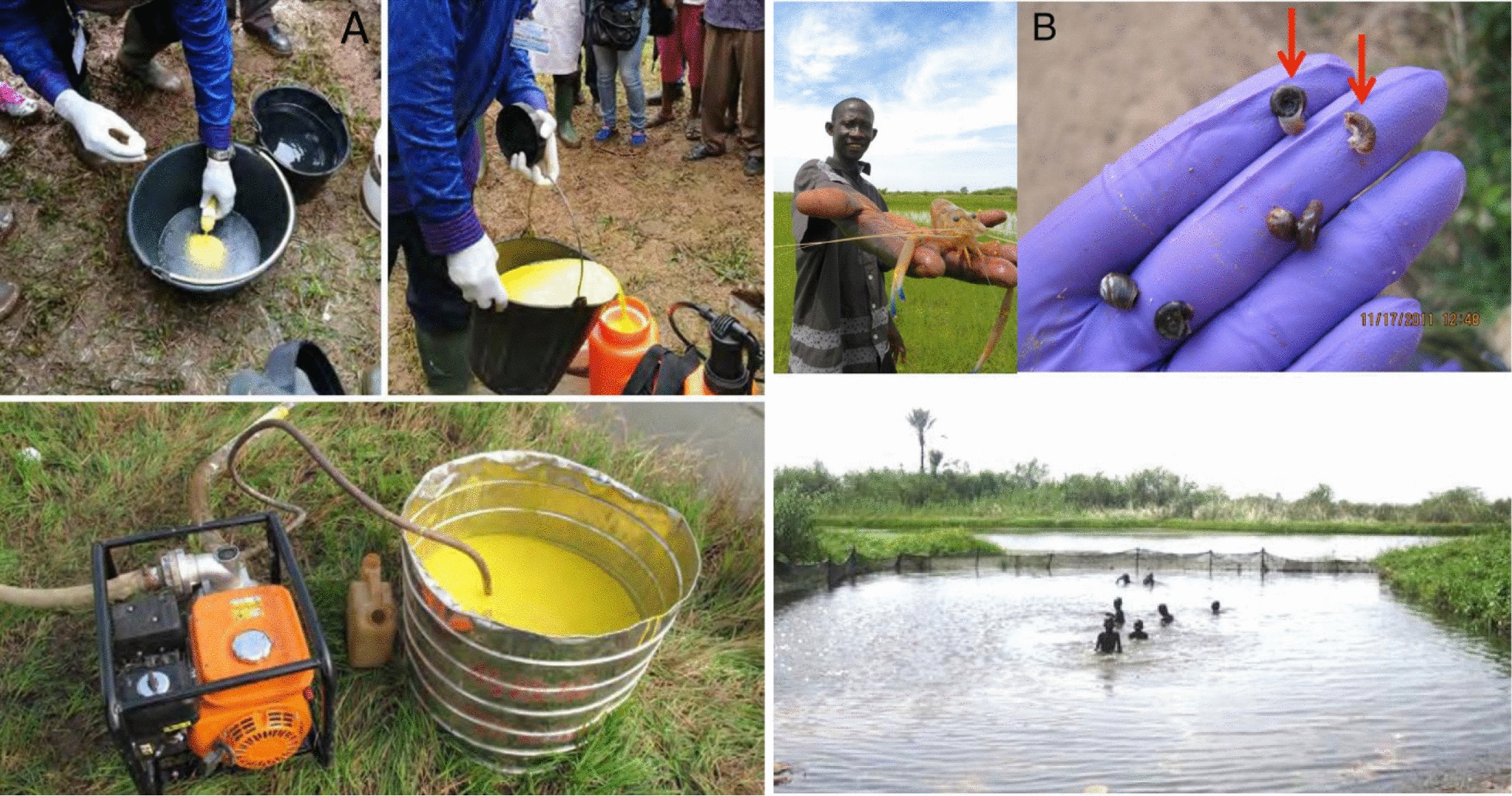
**A** Use of a chemical molluscicide [95] and **B** the predator prawn *Macrobrachium vollenhovenii* [86] against intermediate snail hosts

Niclosamide or bayluscide is a product specially developed to control freshwater snails which act as intermediate hosts for schistosomiasis and other trematodoses such as fascioliasis [86]. It is effective against snails and their eggs, at low concentrations and within a few hours (Fig. 6A). A study carried out in Cameroon showed that niclosamide sensitivity varied according to snail species and population. Egg embryos of *Bi. pfeifferi* were more sensitive than those of *Bu. truncatus*. However, in adults, *Bu. truncatus* was the most sensitive (100% mortality rate) [87]. This chemical also eliminates the free-living stages of the schistosome parasite present in the water (miracidia and cercariae). Niclosamide is, however, harmful to non-target aquatic fauna such as fish and frogs, which would limit its use.

The use of molluscicidal plant products is becoming attractive due to their environmental friendliness, accessibility and ease of application. Mandefro et al. [85] demonstrated that *Achyranthes aspera* (Amaranthaceae), a medicinal plant recognised in many regions of Ethiopia, has molluscicidal effect against to two species of snail, *Bi. pfeifferi* and *L. natalensis*. Similarly, in Egypt, Ibrahim and Abdalla, [88] showed that the aqueous extract of *Moringa oleifera* seeds was toxic to *Bi. alexandrina* snails at a LC_50_ of 0.27 g/l.

Other methods have also been demonstrated, in particular the use of bio-agents predating on intermediate host snails. This is the case of the river shrimp species *Macrobrachium vollenhovenii*, introduced in a field experiment in Senegal as part of a schistosomiasis control programme. Sokolow et al. [89] showed that the abundance of infected snails was 80% lower in the village where *M. vollenhovenii* had been introduced, leading to a 18% ± 5% reduction in the prevalence of human schistosomiasis and 50% ± 8% reduction in the schistosome egg load in the shrimp farming villages compared with the control village (Fig. 6B).

In addition to direct control methods (chemical and biological), indirect methods have also been used. These involve modifying the snails’ environment by destroying their natural habitat, for example by periodically draining open irrigation channels and destroying the vegetation or weeding the riverbanks in order to hinder their development [90].

It is important to note that other trematode or nematode parasites (such as *Angiostrongylus cantonensis*) transmitted by freshwater snails, have not been reported in Africa or remain little studied. This is the case for paragonimosis and clonorchiasis, which are widespread in Asia, where they are endemic in certain regions [91]. These two food-borne diseases are particularly linked to the consumption of raw or poorly cooked fish or crustaceans (the second intermediate host). Further studies of these specific parasitic diseases would be interesting, given that intermediate hosts have been reported in Africa.

## Conclusions

Parasitic diseases such as schistosomiasis and fascioliasis caused by trematodes, involving freshwater snails as intermediate hosts, are present throughout the world, particularly in many African countries. These diseases are of great medical and veterinary importance and represent a heavy public health burden in Africa. The main control strategies for these diseases target the definitive host, although treatment of humans and livestock has been associated with reinfections or the presence of drug-resistant strains. Hence the need to develop strategies for controlling intermediate snail hosts. Indeed, stopping the parasitic development cycle before infection of the definitive vertebrate host appears to be a promising control strategy. However, further studies aimed at improving our currently limited knowledge of the biology of these snails, in particular their ecology and epidemiology, are mandatory before such approaches can be effectively implemented in large-scale control programs. In this respect, the use of modern diagnostic tools, in particular PCR and MALDI-TOF MS, would enable us to better assess the diversity of parasites infecting snails, and to understand snail-parasite relationships in order to refine control strategies directed against snails. However, a limitation of our review is that infection prevalence data in freshwater snails have not been traced in all the studies included. Traditional approaches such as cercarial excretion are widely used and may not only underestimate infection in snail hosts but also increase the risk of parasite misidentification compared with innovative molecular biology or spectrometry tools. Hence further studies or reviews are warranted to shed light on these different aspects.

## Supplementary Information

Supplementary material 1: Fig. S1 Multi-host life cycle of the echinostomid fluke. Unembryonated eggs are passed in feces of infected definitive hosts (1) and develop in water (2). Miracidia usually take about 3 weeks to mature before hatching (3), after which they swim freely and penetrate the first intermediate host, a snail (4). The intramolluscan stages include a sporocyst stage (4a), one or two generations of rediae (4b), and cercariae (4c), which are released from the snail. The cercariae may encyst as metacercariae within the same first intermediate host or leave the host and penetrate a new second intermediate host (5). The definitive host becomes infected after eating metacercariae in infected second intermediate. Hosts (6). Metacercariae excyst in the duodenum (7) and adults reside in the small intestine (for some species, occasionally in the bile ducts or large intestine) (8) [17]. Fig. S2. Distribution of schistosome species in Africa (WHO, https://espen.afro.who.int/regions/who-african-region-afro).

## Data Availability

All relevant data are provided in the manuscript and in these supplementary files.

## Abbreviations

COI: Cytochrome C oxidase
DH: Definitive host
DNA: Deoxyribonucleic Acid
ELISA: Enzyme-Linked Immuno Assay
FSBPD: Freshwater snail-borne parasitic diseases
IH: Intermediate host
LAMP: Loop-mediated amplification
MALDI-TOF MS: Matrix-assisted laser desorption/ionisation time-of-flight mass spectrometry
NTDs: Neglected Tropical Diseases
PCR: Polymerase Chain Reaction
RT-: Real-Time PCR
SSA: Sub-Saharan Africa
WHO: World Health Organization
