# Supplementary figures and images for: Freshwater snail-borne parasitic diseases in Africa
Source: Trop Med Health. 2024 Sep 20;52:61. doi: 10.1186/s41182-024-00632-1 (PMC11414283; doi:10.1186/s41182-024-00632-1)

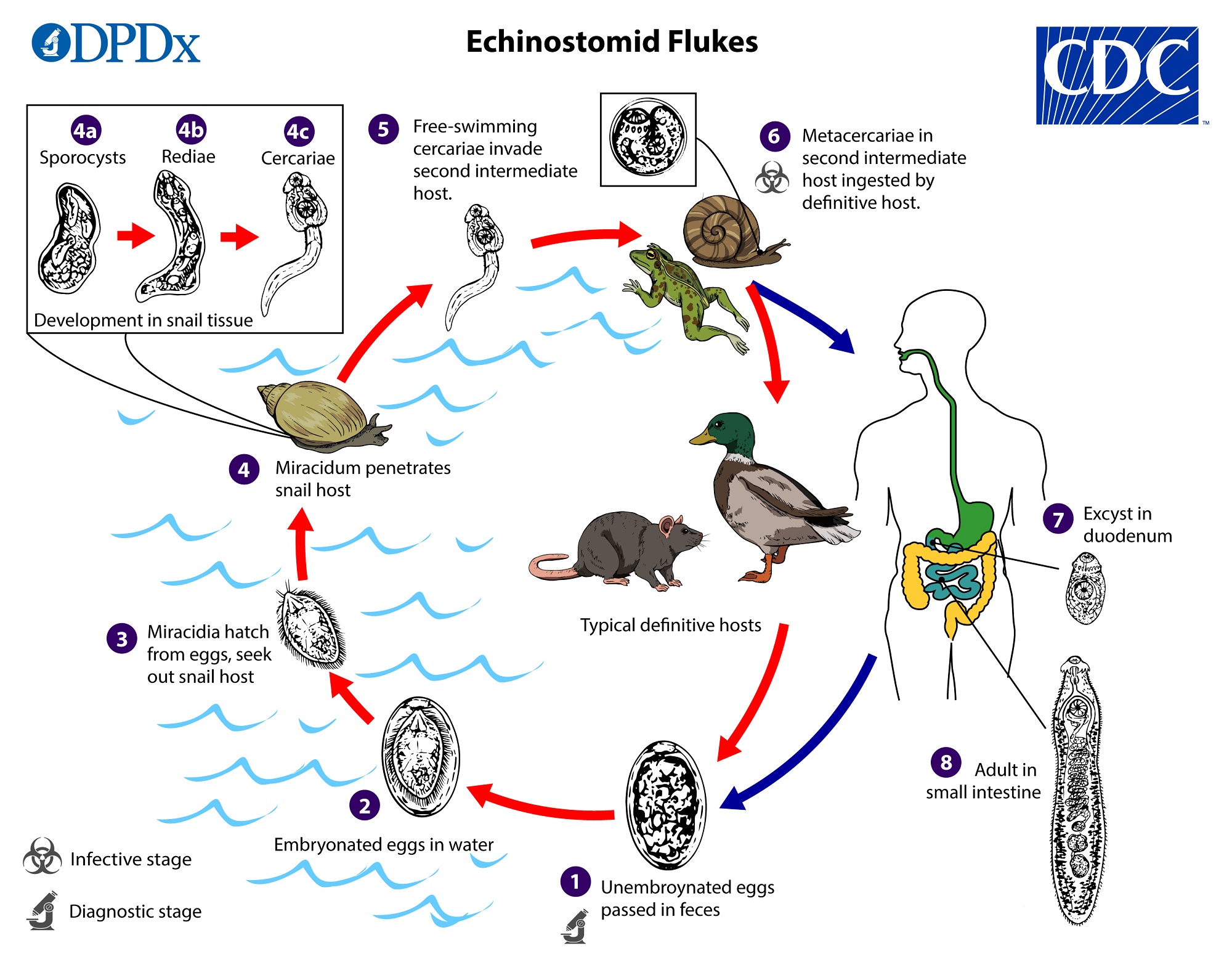

Supplement: Supplementary file 1 — Supplementary material 1: Fig. S1 Multi-host life cycle of the echinostomid fluke. Unembryonated eggs are passed in feces of infected definitive hosts (1) and develop in water (2). Miracidia usually take about 3 weeks to mature before hatching (3), after which they swim freely and penetrate the first intermediate host, a snail (4). The intramolluscan stages include a sporocyst stage (4a), one or two generations of rediae (4b), and cercariae (4c), which are released from the snail. The cercariae may encyst as metacercariae within the same first intermediate host or leave the host and penetrate a new second intermediate host (5). The definitive host becomes infected after eating metacercariae in infected second intermediate. Hosts (6). Metacercariae excyst in the duodenum (7) and adults reside in the small intestine (for some species, occasionally in the bile ducts or large intestine) (8) [17]. Fig. S2. Distribution of schistosome species in Africa (WHO, https://espen.afro.who.int/regions/who-african-region-afro). [file 41182_2024_632_MOESM1_ESM.zip › Supplementary file/Fig. S1.jpg]

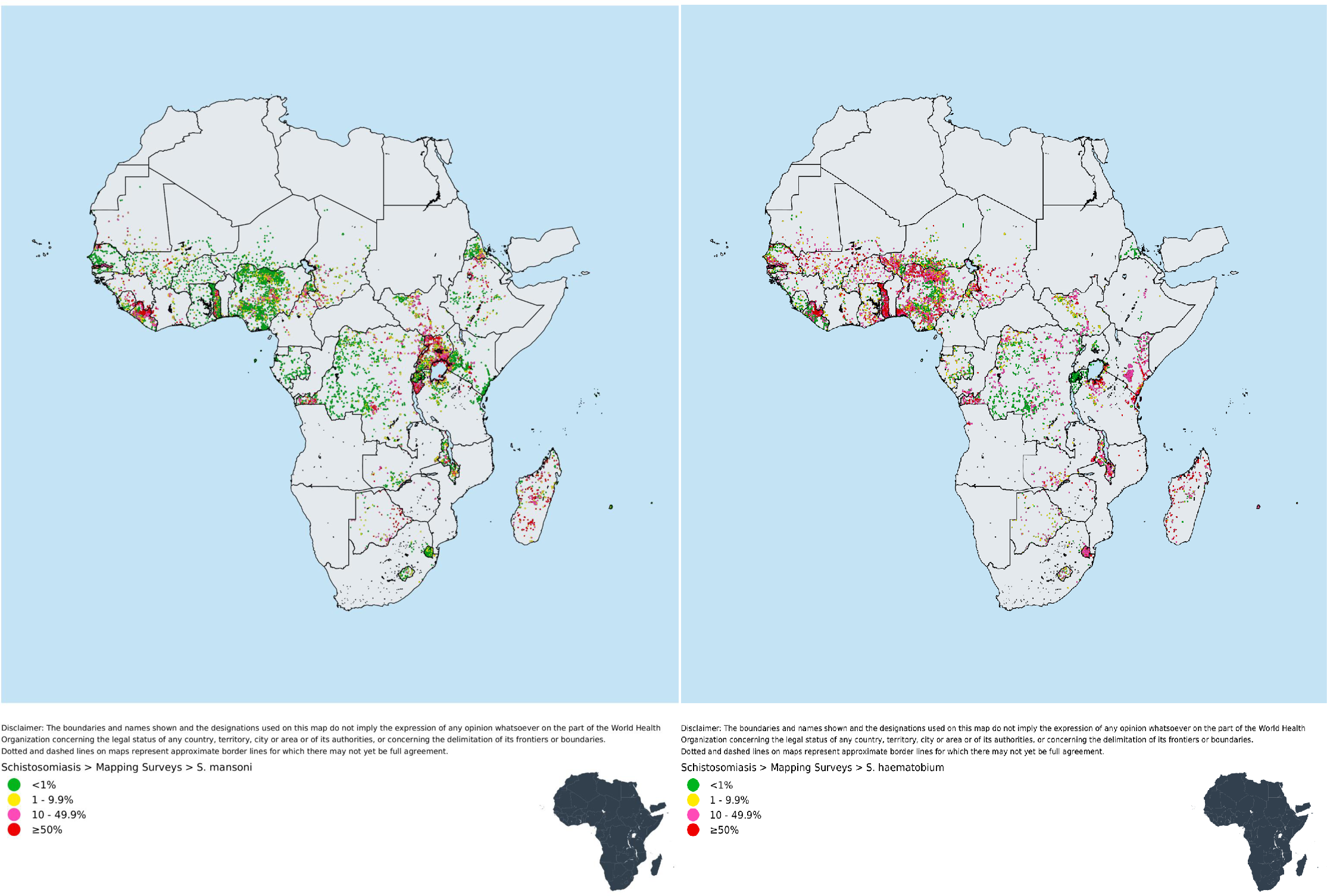

Supplement: Supplementary file 1 — Supplementary material 1: Fig. S1 Multi-host life cycle of the echinostomid fluke. Unembryonated eggs are passed in feces of infected definitive hosts (1) and develop in water (2). Miracidia usually take about 3 weeks to mature before hatching (3), after which they swim freely and penetrate the first intermediate host, a snail (4). The intramolluscan stages include a sporocyst stage (4a), one or two generations of rediae (4b), and cercariae (4c), which are released from the snail. The cercariae may encyst as metacercariae within the same first intermediate host or leave the host and penetrate a new second intermediate host (5). The definitive host becomes infected after eating metacercariae in infected second intermediate. Hosts (6). Metacercariae excyst in the duodenum (7) and adults reside in the small intestine (for some species, occasionally in the bile ducts or large intestine) (8) [17]. Fig. S2. Distribution of schistosome species in Africa (WHO, https://espen.afro.who.int/regions/who-african-region-afro). [file 41182_2024_632_MOESM1_ESM.zip › Supplementary file/Fig. S2.tif]
